# Monitoring clinical and microbiological evolution of a cystic fibrosis patient over 26 years: experience of a Brazilian CF Centre

**DOI:** 10.1186/s12890-017-0442-2

**Published:** 2017-07-14

**Authors:** Cassiana da Costa Ferreira Leite, Tania Wrobel Folescu, Mônica de Cássia Firmida, Renata Wrobel Folescu Cohen, Robson Souza Leão, Flávia Alvim Dutra de Freitas, Rodolpho Mattos Albano, Claudia Henrique da Costa, Elizabeth Andrade Marques

**Affiliations:** 1 Departamento de Microbiologia, Imunologia e Parasitologia, Faculdade de Ciências Médicas, Universidade do Estado do Rio de Janeiro, Rio de Janeiro, Brazil; 2 Instituto Nacional de Saúde da Mulher da Criança e do Adolescente Fernandes Figueira, Fundação Oswaldo Cruz, Rio de Janeiro, Brazil; 3 Departamento de Doenças do Tórax, Faculdade de Ciências Médicas, Universidade do Estado do Rio de Janeiro, Rio de Janeiro, Brazil; 4 Departamento de Bioquímica, Instituto de Biologia Roberto Alcântara Gomes, Universidade do Estado do Rio de Janeiro, Rio de Janeiro, Brazil; 5 Departamento de Microbiologia, Imunologia e Parasitologia, Faculdade de Ciências Médicas, Universidade do Estado do Rio de Janeiro, Brazil Av. 28 de setembro 87, Fundos, Terceiro andar– Vila Isabel, Rio de Janeiro, RJ Brazil

**Keywords:** *Burkholderia vietnamiensis*, *Burkholderia cepacia* complex, Cystic fibrosis, Microbiome, Chronic lung disease, Cystic fibrosis-related diabetes

## Abstract

**Background:**

*Burkholderia cepacia* complex is a group of opportunistic pathogens in cystic fibrosis (CF) patients believed to be associated with poor prognosis and patient-to-patient transmissibility. Little is known about clinical outcomes after *B. vietnamiensis* chronic colonization/infection.

**Case presentation:**

A 33 yo male patient had diagnosis of CF by 7 yo, after recurrent pneumonia during infancy and lobectomy (left upper lobe) at 6 yo. *Burkholderia cepacia* complex (Bcc) was first isolated by 13 yo, and the patient fulfilled the criteria for chronic colonization by 15 yo. In the following 16 years (1997–2013), there was intermittent isolation of *P. aeruginosa* and continuous isolation of Bcc, identified as *B. vietnamiensis*. There was clinical and laboratorial stability for 16 years with annual rate of decline in forced expiratory volume in 1 s (FEV1) and forced vital capacity (FVC) of 1.61 and 1.35%, respectively. From 2013 to 2015, there was significant clinical and lung function deterioration: annual rate of decline in FEV1 and FVC was 3 and 4.1%, respectively while body mass index decreased from 18.1 to 17.1. Episodes of hemoptysis and respiratory exacerbations (with hospital admissions) became more frequent. CF related diabetes was diagnosed (fasting glycemia: 116 mg/dL, oral glucose tolerance test: 305 mg/dL). Because of the severity of the disease in the last years, in addition to traditional microbiological surveillance, microbiome analysis by next generation sequencing (NGS) was performed on respiratory secretions. The NGS showed that 97% of the sequencing data were attributed to genus *Burkholderia.*

**Conclusions:**

We report the case of a 33-year-old male CF patient known to have chronic infection with *B. vietnamiensis* who remained clinically stable for 16 years and presented recent clinical and laboratorial deterioration. Microbiome analysis of respiratory secretions was performed in 3 samples collected in 2014–2015. Clinical deterioration overlapped with cystic fibrosis-related diabetes and microbiome composition revealed no significant differences when compared microbiome results to culture dependent methods.

## Background

*Burkholderia cepacia* complex (Bcc) is a group of opportunistic pathogens, with at least 20 distinct genomovars/species [1], believed to be associated with poor prognosis and patient-to-patient transmissibility in cystic fibrosis (CF). *Burkholderia cenocepacia* and *B. multivorans* are more prevalent, but in Brazil *B. vietnamiensis* was identified as the second most prevalent species in this species complex in patients attending a reference center [2].

In comparison with *B. cenocepacia*, the clinical significance of a chronic colonization of the airways by the other Bcc species is much less clear. The chronic airway colonization by *B. multivorans*, for example, has not been associated with an increased mortality or accelerated decline in the lung function when compared to chronic colonization with *Pseudomonas aeruginosa* [3–5].

Based on these considerations, we describe the 26 year follow-up of a CF patient colonized with *B. vietnamiensis* who remained clinically stable for 16 years and experienced progressive progressive clinical worsening in the last 3 years. This case raised several issues regarding factors influencing clinical deterioration and the possible impact of changes in lung microbiome composition.

## Case presentation

A 33-year-old male patient had diagnosis of CF by 7 yo attested by two positive sweat chloride tests (136.6 m Eq/L and 129.8 mEq/L) and sequence analysis of the gene *CFTR* (homozygosity for G85E). Before this time, he had recurrent pneumonia during infancy and was submitted to lobectomy (left upper lobe) at 6 yo.

**Fig. 1:**
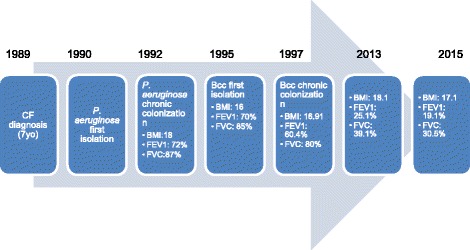
Timeline

After diagnosis, he presented chronic sinopulmonary disease characterized by chronic cough, recurrent wheezing and gastrointestinal impairment with pancreatic insufficiency and failure to thrive. *Pseudomonas aeruginosa* was first isolated in sputum samples by 8 yo (1990). Chronic colonization by mucoid and non mucoid *P. aeruginosa* was recognized at 10 yo (1992). Three years later (13 yo - 1995), Bcc was first isolated, and the patient fulfilled the criteria for Bcc chronic colonization [6] by 15 yo. In the following 16 years (1997–2013), there was intermittent isolation of *P. aeruginosa* and continuous isolation of Bcc, identified as *B. vietnamiensis* [7] (Fig. 1). Clinical data were registered during each visit at the CF center, including body mass index (BMI) and spirometric measurements (% predicted), i.e. forced expiratory volume in 1 s (FEV_1_) and forced vital capacity (FVC). In order to study the trend of respiratory function parameters over time, it was calculated the annual rate of decline by linear regression.

At the moment of chronic *P. aeruginosa* colonization, the patient presented: BMI,18; FEV_1_, 72%; and FVC, 87%. At the moment of the first isolation of Bcc, BMI, FEV_1_ and FVC were of 16, 70 and 85%, respectively. These values changed to 16.91, 64 and 80% for BMI, FEV_1_ and FVC when chronic Bcc isolation was identified. From 1995 to 2013, the patient remained clinically stable, using regular medication for CF and inhaled antibiotics for *Pseudomonas aeruginosa* chronic colonization (tobramycin alternate months). He required on average one hospital admission per year due to respiratory exacerbation and, during this period, presented BMI:16.9–18.1, with FEV_1_ (%) and FVC (%) annual rate of decline of 1.61 and 1.35%, respectively.

However, from 2013 to 2015, there was significant clinical and lung function deterioration: FEV_1_% and FVC% annual rate of decline was 3 and 4.1% respectively while BMI decreased from 18.1 to 17.1. He presented glucose intolerance in 2013, with lung function close to this moment showing FEV_1_ of 25.1% and FVC of 39.1%. Since June 2014, he started episodes of hemoptysis and required more frequent hospital admissions due to respiratory exacerbations. CF related diabetes (CFRD) was diagnosed in 2014 (fasting glycemia: 116 mg/dL, oral glucose tolerance test: 305 mg/dL). The patient was on regular use of alpha dornase, short and long action inhaled bronchodilators, inhaled corticosteroids, azithromycin, pancreatic enzymes and insulin replacement. By this time, the patient showed poor adherence to treatment and received oral ciprofloxacin for 14 days (June, 2014) and IV meropenem (September, 2014) during hospitalization. In the following year (2015), he was admitted in hospital due to respiratory exacerbation twice.

**Fig. 2:**
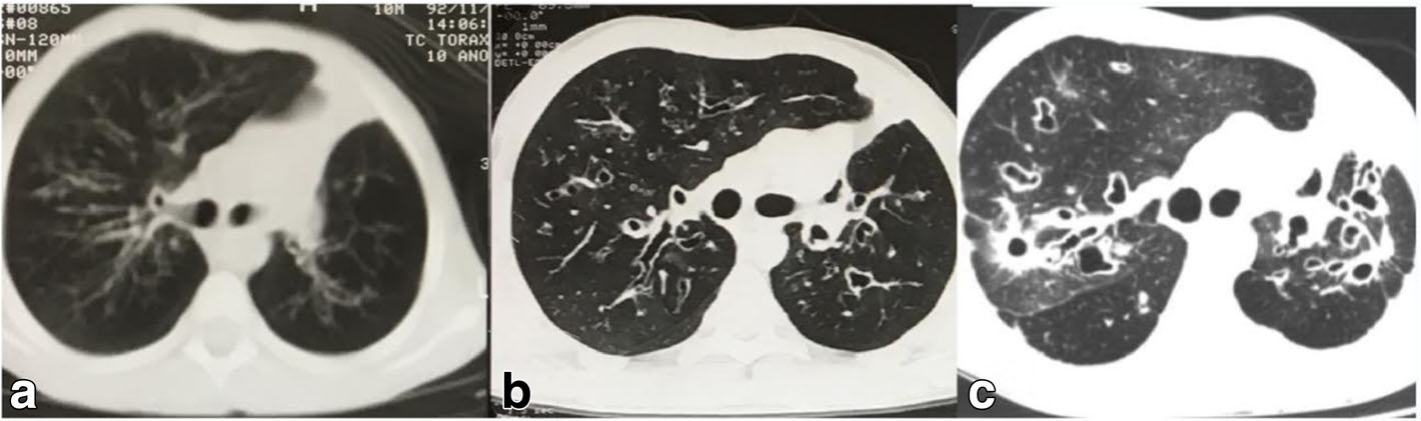
High resolution computed tomography images: 1992 (**a**); 2000 (**b**); 2015 (**c**)

Available high resolution computed tomography (HRCT) evaluation through modified Bhalla score [8] presented the following total score: 9 in 1992, 17 in 2000 and 25 in 2015. The last HRCT scan (2015) highlighted the increase in severity of bronchiectasis, thickening of bronchial walls, mosaic pattern, air trapping and diffuse ground glass images (Fig. 2).

Because of the severity of the disease in the last years (2014–2015), in addition to traditional microbiological surveillance, microbiome analysis by next generation sequencing was performed on respiratory secretions at three clinical visits (June 27, 2014; September 26, 2014; and January 09, 2015). Aerobic cultures from these sputum samples presented two main microorganisms related to lung infection/colonization in the CF patient: *P. aeruginosa* and Bcc (identified as *B. vietnamiensis* by polymerase chain reaction (PCR) and sequencing of the *rec*A gene). The first two samples presented Bcc and *P. aeruginosa* (non mucoid type), while *P. aeruginosa* (non mucoid type) and Bcc with both mucoid and non mucoid morphotypes were identified in the third sample.

Microbiome analysis of sputum samples was performed by sequencing the V4 region of the 16S rRNA gene in an Illumina MiSeq instrument [9]. The following data was obtained from the three samples collected from June 2014 to January 2015 (6 months period): 97% of the sequencing data were attributed to genus *Burkholderia* (98.7, 98.6 and 92.3% of each sputum sample, respectively). Although *Burkholderia* was remarkably predominant, other genera related to CF lung infections were also found: *Pseudomonas* (0.71%), *Rothia* (1.65%), *Staphylococcus* (0.07%), *Haemophilus* (0.29%), *Ralstonia* (0.002%) (Table 1).

**Table 1:** Presence in aerobic cultures and microbiome analysis of collected sputum samples

	June 2014		September 2014		January 2015	
	Presence in Culture	Microbiome Analysis	Presence in Culture	Microbiome Analysis	Presence in Culture	Microbiome Analysis
*Burkholderia*	Yes	98.7%^a^	Yes	98.6%	Yes	92.6%
*Rothia*	No	0.53%	No	0.67%	No	4.6%
*Pseudomonas*	Yes	0.57%	Yes	0.47%	Yes	1.27%
Unclassified Enterobacteriaceae	No	0%	No	0%	No	0.04%
Unlcassified Burkholderiaceae	-	0%	-	0.02%	-	0%
Unclassified Burkholderiales	-	0%	-	0%	-	0.25%
*Ralstonia*	No	0%	No	0.006%	No	0%
Unclassified Alcaligenaceae	-	0%	-	0.006%	-	0.02%
*Haemophilus*	No	0%	No	0%	No	1.12%
*Staphylococcus*	No	0.08%	No	0.04%	No	0.13%

^a^Relative proportion

## Discussion and conclusions

Despite advances in Bcc taxonomy, predicting prognosis in infected CF patients is challenging. Previous studies have shown that CF patients chronically colonized with Bcc present greater deterioration of lung function, require frequent antibiotic therapy and display increased mortality when compared to patients colonized with *P. aeruginosa* [10, 11]. However, no studies have described the clinical evolution of CF patients chronically colonized with *B. vietnamiensis*. In our case, a CF patient chronically colonized with *B. vietnamiensis* remained in stable conditions for over than 16 years, with FEV1 and FVC annual rate of decline within the expected range, and experienced significant clinical deterioration in the last 3 years: he presented hemoptysis, lung function decline and more exacerbations, despite regular prescription of maintenance therapy.

For CF lung disease, risk factors associated with FEV_1_ decline include (but are not limited to) young age, high lung function, female gender, modifier genes, pancreatic insufficiency, poor nutritional status, viral respiratory infections, colonization with *P. aeruginosa* and Bcc, and diabetes mellitus [12]. Mean rate of FEV_1_% predicted decline in CF patients chronically colonized with Bcc was significantly higher than expected in all ages [6], although there was no correlation to Bcc species. In our report, chronic Bcc colonization and clinical stability had been present over a long period. However, the lung function steadly declined in the last 3 years, coinciding with the moment of CFRD diagnosis. CFRD is an important cause of morbidity in CF since insulin insufficiency and hyperglycemia, promoting oxidative stress, inflammation and infection, negatively affect CF lung disease. Nutritional status and pulmonary function begin to decline in CF patients several years before the actual diagnosis of CFRD, when minimal hyperglycemia is present. Abnormal glucose tolerance relates to insulin insufficiency and insulin resistance [13]. Since insulin is an anabolic hormone, insulin insufficiency may reduce inspiratory and diaphragm muscle function, further worsening lung function [14]. The deterioration of the reported CF patient lung function overlaps with CFRD diagnosis and, in fact, hyperglycemia may have started before and contributed to clinical deterioration as well as other factors such as poor adherence to treatment. In addition to all clinical factors that might justify clinical deterioration, culture-independent techniques could be of use to allow a greater understanding of the respiratory microbiome in this CF patient. This analysis confirmed that *Burkholderia* was the predominant genus. *Pseudomonas* appeared in low relative abundance in the three sputum samples, suggesting that the status of *P. aeruginosa* on routine microbiological cultures, usually referred as the major CF pathogen, could be overstated. In conclusion, clinical factors such as CFRD and poor adherence to treatment may have contributed to lung function deterioration. Nevertheless, although less frequent than the bacterial infections, we cannot exclude the fungal and viral respiratory infections as a risk factor associated with FEV_1_ decline. In addition, despite the small number of samples used for culture-independent analysis which limits our understanding of the lung microbiome of this patient, there was a good agreement with sputum cultures that showed chronic colonization with Bcc.

## Abbreviations

Bcc: *Burkholderia cepacia* complex
BMI: Body mass index
CF: Cystic fibrosis
CFRD: CF-related diabetes
FEV1: Forced expiratory volume in 1 s
FVC: Forced vital capacity
HRCT: High-resolution computed tomography
PCR: Polymerase chain reaction
